# Simplification of the Gram Matrix Eigenvalue Problem for Quadrature Amplitude Modulation Signals

**DOI:** 10.3390/e24040544

**Published:** 2022-04-13

**Authors:** Ryusuke Miyazaki, Tiancheng Wang, Tsuyoshi Sasaki Usuda

**Affiliations:** 1 Graduate School of Information Science and Technology, Aichi Prefectural University, Nagakute 480-1198, Aichi, Japan; im201011@cis.aichi-pu.ac.jp; 2 Faculty of Engineering, Kanagawa University, Yokohama 221-8686, Kanagawa, Japan

**Keywords:** quantum communication, quantum cipher, quadrature amplitude modulation (QAM), coherent state, Gram matrix, square-root measurement (SRM)

## Abstract

In quantum information science, it is very important to solve the eigenvalue problem of the Gram matrix for quantum signals. This allows various quantities to be calculated, such as the error probability, mutual information, channel capacity, and the upper and lower bounds of the reliability function. Solving the eigenvalue problem also provides a matrix representation of quantum signals, which is useful for simulating quantum systems. In the case of symmetric signals, analytic solutions to the eigenvalue problem of the Gram matrix have been obtained, and efficient computations are possible. However, for asymmetric signals, there is no analytic solution and universal numerical algorithms that must be used, rendering the computations inefficient. Recently, we have shown that, for asymmetric signals such as amplitude-shift keying coherent-state signals, the Gram matrix eigenvalue problem can be simplified by exploiting its partial symmetry. In this paper, we clarify a method for simplifying the eigenvalue problem of the Gram matrix for quadrature amplitude modulation (QAM) signals, which are extremely important for applications in quantum communication and quantum ciphers. The results presented in this paper are applicable to ordinary QAM signals as well as modified QAM signals, which enhance the security of quantum cryptography.

## 1. Introduction

The efficient computations and evaluations of quantities such as the error probability, mutual information, channel capacity, and reliability function are extremely important in quantum communication, quantum radar, and quantum cipher systems [1,2,3,4,5]. The computation of these quantities is essential not only for evaluating the reliability of quantum communication and the sensitivity of quantum radar but also for guaranteeing the security of quantum cryptography. In particular, because the security of a quantum stream cipher relies on the difference between the quantum optimum receiving capabilities of the legitimate receiver and the eavesdropper, it is essential to evaluate the optimum quantum receiver performance of the eavesdropper to guarantee security [6,7]. In quantum stream ciphers, the number of signals usually runs to several hundreds or thousands [8,9]. However, recent experiments have shown that some cases may contain millions or even billions of signals [10,11].

The eigenvalues and eigenvectors of the Gram matrix are very useful for computing various quantities that evaluate system performance. By solving the eigenvalue problem of the Gram matrix and finding its square root, the channel matrix given by the so-called square-root measurement (SRM) [12,13,14,15,16] can be computed. This implies that the error probability and mutual information using SRM can be directly calculated. SRM is asymptotically optimal for any quantum state signals with respect to minimizing the error probability, and it is used in the proof of the quantum channel coding theorem [14]. Moreover, SRM is strictly optimal for symmetric pure-state signals with uniform *a priori* probabilities [12,13,14,15,17,18,19]. Actually, SRM is also strictly optimal for some asymmetric pure-state signals with not necessarily uniform *a priori* probabilities [20]. As each component of the square root of the Gram matrix corresponds to the inner product of a signal quantum state and a measurement state of the SRM, a matrix representation of the signal quantum state can be obtained when the signal quantum states are linearly independent [21]. This representation is known to be useful for analyzing quantum systems (e.g., [21]). Furthermore, even if the quantum state is a vector in an infinite-dimensional Hilbert space, such as a coherent state or squeezed state, the matrix form allows numerical calculations to be performed because it provides a representation in a finite-dimensional subspace (e.g., [22]). Because the Gram matrix is a matrix representation of the density operator of the quantum information source, the Holevo capacity [14] and the upper and lower bounds of the reliability function [23,24] can be directly calculated by using its eigenvalues.

In general, the Gram matrix is M×M for *M*-ary pure-state signals. Therefore, if we use a universal numerical algorithm to compute the eigenvalues and eigenvectors of the Gram matrix, the computation is hard when *M* is large. However, if the signals are symmetric, the analytic solutions of the Gram matrix eigenvalues and eigenvectors can be obtained by using well-known operations in linear algebra. In addition, by using the character [25] of a group, analytic solutions [26] can be obtained for narrow-sense group covariant signals [27], which are a generalization of symmetric signals. Narrow-sense group covariant signals are important in applications such as phase-shift keying (PSK) coherent-state signals and coded symmetric signals. Unfortunately, however, several important asymmetric signals are not narrow-sense group covariant, such as amplitude-shift keying (ASK) coherent-state signals and quadrature amplitude modulation (QAM) coherent-state signals [27]. QAM coherent-state signals are extremely important for quantum communication [28] and quantum ciphers [29]; moreover, QAM signals almost achieve the quantum channel capacity under energy constraints [30].

Recently, we showed that the eigenvalue problem of the Gram matrix can be simplified by using its partial symmetry for ASK coherent-state signals and amplitude-modulated phase-modulated (AMPM) signals, which belong to a class of asymmetric signals [31,32,33]. In this paper, we show that the eigenvalue problem of the Gram matrix can also be simplified by using its partial symmetry for QAM signals, which are more important for applications than ASK and AMPM signals. The method in this paper is applicable to ordinary QAM signals as well as modified QAM signals, which enhance the security of quantum stream ciphers [29]. Note that the signals considered in this paper belong to a class of asymmetric signals defined in Ref. [20], where the class is referred to as “the multiple constellations of geometrical uniform symmetry (GUS) state”. The results of this paper are closely related to Ref. [20].

The remainder of this paper is organized as follows. In Section 2, we introduce some preliminaries and basic theory. First, we define quantum signals and measurements, and then we explain various quantities such as the error probability, mutual information, and Holevo capacity. Next, we introduce the Gram matrix, SRM, and symmetric signals, which are the subject of this paper. In Section 3, we present the main results. For the eigenvalue problem of the Gram matrix of M=4m QAM signals, we show that the size of the problem can be reduced by using the partial symmetry of the signals. In Section 4, we show examples for the simplest case of m=2 and provide specific forms of eigenvalues and eigenvectors for the smaller matrices than the Gram matrix. In Section 5, we provide numerical experiments as examples of applications for the main result. Finally, in Section 6, we summarize the conclusions to this study.

## 2. Basic Theory

### 2.1. Quantum Signals and Measurements

Let H be the Hilbert space of a quantum system. The set of *M*-ary pure-state signals is represented by the following:

(1) $$S=\{|\psi_{i}\rangle \in H\;|\;i=1,2,\ldots ,M\},$$

where $\langle \psi_{i}|\psi_{i}\rangle =1$. Let $\xi_{i}$ be the *a priori* probability of state $|\psi_{i}\rangle$. Then, the pair (S,ξ) is referred to as a quantum information source or a quantum ensemble.

In general, a quantum measurement is mathematically described by a positive operator-valued measure (POVM). The POVM is described as follows:

(2) $$\Pi =\{{\hat{\Pi }}_{j}\;|\;j=1,2,\ldots ,M\},$$

where $\hat{\Pi }$ is a Hermitian operator on H satisfying the following.

$$\hat{\Pi }\geq 0,\;\sum_{j=1}^{M}\hat{\Pi }=\hat{I}.$$

Here, $\hat{I}$ is the identity operator on H. Although POVM is a mathematical representation of a quantum measurement, it may be called a quantum measurement. The conditional probability that the result *j* is obtained when performing the measurement Π on quantum state $|\psi_{i}\rangle$ is as follows.

(3) $$P\left(j|i\right)=\;\mathrm{Tr}\;\left(|\psi_{i}\rangle \langle \psi_{i}|{\hat{\Pi }}_{j}\right).$$

### 2.2. Error Probability, Mutual Information, and Holevo Capacity

Suppose we measure the quantum information source (S,ξ) by a POVM Π. Using Equation (3), the average error probability is defined as follows:

(4) $$P_{e}=\sum_{i=1}^{M}\xi_{i}\sum_{j\neq i}P\left(j|i\right)=1-\sum_{i=1}^{M}\xi_{i}P\left(i|i\right),$$

which is also simply called the error probability. Then, the following is the case:

(5) $$P_{e}^{\left(\mathrm{opt}\right)}=\min_{\Pi }P_{e}$$

and it is referred to as the minimum error probability and the set Π that attains $P_{e}^{\left(\mathrm{opt}\right)}$ is called the optimum POVM. The mutual information is defined as follows:

(6) $$I\left(S,\xi \right)=\sum_{i=1}^{M}\xi_{i}\sum_{j=1}^{M}P\left(j|i\right)\log_{2}\left[\frac{P(j|i)}{\sum_{k=1}^{M}\xi_{k}P\left(j|k\right)}\right],$$

and its maximization with respect to quantum measurements is the following:

(7) $$I_{\mathrm{acc}}=\max_{\Pi }I\left(S,\xi \right),$$

which is called accessible information. For (S,ξ), the following is the case:

(8) $$\hat{\rho }=\sum_{i=1}^{M}\xi_{i}|\psi_{i}\rangle \langle \psi_{i}|$$

and it is called the density operator of the quantum information source. Using the density operator, we define von Neumann entropy as follows.

(9) $$\chi \left(\xi \right)=-\mathrm{Tr}\left(\hat{\rho }\log_{2}\hat{\rho }\right).$$

When the signals are pure states, the maximization of χ(ξ) with respect to ξ is the so-called Holevo capacity.

(10) $$C=\max_{\xi }\chi \left(\xi \right).$$

Let $\lambda_{j}$ be the eigenvalues of $\hat{\rho }$ corresponding to the ξ that attains *C*. Then, the Holevo capacity can be calculated as follows.

(11) $$C=-\sum_{j}\lambda_{j}\log_{2}\lambda_{j}.$$

The error probability and mutual information and their optimal values are calculated using the conditional probability (3), while the Holevo capacity uses the density operator (8) of the quantum information source.

### 2.3. Gram Matrix

For an *M*-ary pure-state signal set $S=\{|\psi_{i}\rangle \;|\;i=1,2,\ldots ,M\}$, the Gram matrix Γ is defined as follows.

(12) $$\begin{array}{c} \Gamma =\left[\begin{array}{cccc} \langle \psi_{1}|\psi_{1}\rangle & \langle \psi_{1}|\psi_{2}\rangle & \ldots & \langle \psi_{1}|\psi_{M}\rangle \\ \langle \psi_{2}|\psi_{1}\rangle & \langle \psi_{2}|\psi_{2}\rangle & \ldots & \langle \psi_{2}|\psi_{M}\rangle \\ \vdots & \vdots & \ddots & \vdots \\ \langle \psi_{M}|\psi_{1}\rangle & \langle \psi_{M}|\psi_{2}\rangle & \ldots & \langle \psi_{M}|\psi_{M}\rangle \end{array}\right]. \end{array}$$

The Gram matrix is an M×M matrix in which the (i,j)-th element is the inner product $\langle \psi_{i}|\psi_{j}\rangle$ between quantum state signals (Note that the Gram matrix is sometimes defined by using the inner products between weighted quantum state signals [20,34]). By definition, the Gram matrix is Hermitian; moreover, it is non-negative [35]. Because the norm of the quantum state vector is unity, so are all diagonal components of the Gram matrix, and the sum of the diagonal components is *M*. The Gram matrix is very useful in the theoretical treatment of *M*-ary pure-state signal systems. First, for a quantum information source $\left(S,\left\{\frac{1}{M}\right\}\right)$ for which its *a priori* probabilities are uniform, $\frac{1}{M}\Gamma$ is a matrix representation of its density operator. That is, $\hat{\rho }$ and $\frac{1}{M}\Gamma$ are isomorphic.

(13) $$\hat{\rho }≅\frac{1}{M}\Gamma .$$

In this case, the eigenvalues of the Gram matrix and those of the density operator are identical, and the von Neumann entropy can be calculated using the eigenvalues of the Gram matrix. For symmetric signals, the Holevo capacity can be calculated directly from the eigenvalues of the Gram matrix, because the Holevo capacity is attained with uniform *a priori* probabilities [36]. A similar statement can be made for the upper and lower bounds of the quantum reliability function [37,38]. Furthermore, the Gram matrix is closely related to the theory of SRM, as described below.

### 2.4. Square-Root Measurement

The SRM is a quantum measurement defined using the quantum states that are being transmitted. For a set of *M*-ary pure-state signals $S=\{|\psi_{i}\rangle \;|\;i=1,2,\ldots ,M\}$, the POVM of the SRM $\left\{{\hat{\Pi }}_{j}^{\left(\mathrm{SRM}\right)}\;|\;j=1,2,\ldots ,M\right\}$ is defined as follows:

(14) $$\begin{array}{c} {\hat{\Pi }}_{j}^{\left(\mathrm{SRM}\right)}=|\mu_{j}\rangle \langle \mu_{j}|, \end{array}$$

(15) $$\begin{array}{c} |\mu_{j}\rangle ={\hat{\Psi }}^{-\frac{1}{2}}|\psi_{j}\rangle , \end{array}$$

(16) $$\begin{array}{c} \hat{\Psi }=\sum_{i=1}^{M}|\psi_{i}\rangle \langle \psi_{i}|, \end{array}$$

where vector $|\mu_{j}\rangle$ is the measurement state or measurement quantum state (e.g., [4]). For linearly independent signal systems, the set of measurement quantum states $\left\{|\mu_{j}\rangle \right\}$ is an orthonormal system and is an orthonormal basis of the space spanned by signal quantum states [34]. Although SRM appeared in papers in the 1970s (e.g., Belavkin [12] and earlier papers by Holevo), the name SRM has only been used since 1996, when Hausladen et al. presented the quantum channel coding theorem [14]. They proved that the inner product between quantum states $|\psi_{i}\rangle$ and $|\mu_{j}\rangle$ in Equation (15) is equal to the (i,j)-th element of the square root of the Gram matrix, Γ, and called this the “square-root” measurement. Specifically, they showed the following.

(17) $$\begin{array}{c} \langle \psi_{i}|\mu_{j}\rangle ={\left(\Gamma^{\frac{1}{2}}\right)}_{i,j}. \end{array}$$

The existence of $\Gamma^{\frac{1}{2}}$ is always guaranteed because the Gram matrix is non-negative and Hermitian, as mentioned above. Therefore, Equation (17) denotes a component of the matrix representation of the signal quantum state $|\psi_{i}\rangle$ using the orthonormal basis $\left\{|\mu_{j}\rangle \right\}$. Thus, as the signal quantum state can be represented in matrix form based on the square-root of the Gram matrix, computing $\Gamma^{\frac{1}{2}}$ is very useful for simulating systems such as quantum communication, quantum radar, and quantum ciphers. From Equation (3), we have the following.

(18) $$\begin{array}{cc} P(j|i) & =\;\mathrm{Tr}\;\left(|\psi_{i}\rangle \langle \psi_{i}|{\hat{\Pi }}_{j}^{\left(\mathrm{SRM}\right)}\right)=\;\mathrm{Tr}\;\left(|\psi_{i}\rangle \langle \psi_{i}|\mu_{j}\rangle \langle \mu_{j}|\right)\\ \; & =|\langle \psi_{i}|\mu_{j}\rangle {|}^{2}={\left|{\left(\Gamma^{\frac{1}{2}}\right)}_{i,j}\right|}^{2}. \end{array}$$

Because the matrix in which the (i,j)-th elements are equal to P(j|i) is the channel matrix and obtaining P(j|i) allows the error probability and mutual information to be calculated using Equations (4) and (6). Therefore, if the square root of the Gram matrix can be computed efficiently, it is easy to compute the error probability and mutual information when SRM is applied. In general, the square root of a matrix can be computed using its eigenvalues and eigenvectors. Thus, being able to efficiently compute the eigenvalues and eigenvectors of a Gram matrix is extremely important.

### 2.5. Coherent-State Signals

Coherent states are the most fundamental optical quantum states used in macroscopic quantum communication or quantum ciphers. They are the stable states of light that can be realized by an ideal laser. The coherent state |α〉 with the complex amplitude α is given by the following:

(19) $$\begin{array}{c} |\alpha \rangle =\exp\left(-\frac{{\left|\alpha \right|}^{2}}{2}\right)\sum_{n=0}^{\infty }\frac{\alpha^{n}}{\sqrt{n!}}|n\rangle , \end{array}$$

where |n〉 is the photon number state, and *n* is the number of photons. The inner product between two coherent states |α〉 and |β〉 is as follows:

(20) $$\begin{array}{c} \langle \alpha |\beta \rangle =\exp\left(\alpha^{*}\beta -\frac{{\left|\alpha \right|}^{2}}{2}-\frac{{\left|\beta \right|}^{2}}{2}\right), \end{array}$$

where * denotes complex conjugation. If α and β are both real numbers, the value of 〈α|β〉 is real. In this paper, we assume that the signal quantum state is a coherent state.

Note that the coherent state is completely characterized by its complex amplitude α, as shown in Equation (19). A complex number α is graphically described by a point on the complex plane, and so a coherent state signal is also described by a point on the complex plane. In this case, the complex plane is often called the phase plane.

### 2.6. Symmetric Signals

In the field of quantum information science, Davies defined a group covariant signal [39] with symmetry corresponding to the symmetry of the group, which is sometimes simply called a symmetric signal. Although Davies’ definition of group covariant signals applies to a broader class of signals than the pure-state signals treated in this paper, we adopt the following narrow definition of group covariant signals [27], which is applicable only to simpler pure-state signals.

**Definition** **1** (Narrow-sense group covariant signals [27])**.** *Let (G;∘) be a finite group with the operation *∘*. A set $\left\{|\psi_{i}\rangle |i\in G\right\}$ of quantum state signals is called (narrow-sense) group covariant with respect to the group (G;∘) if the following is the case:*

(21) $$\begin{array}{c} \forall i,k\in G,\;\;\exists {\hat{U}}_{k},\;\;{\hat{U}}_{k}|\psi_{i}\rangle =|\psi_{k\circ i}\rangle , \end{array}$$

*where ${\hat{U}}_{k}$ is a unitary operator.*

Narrow-sense group covariant signals have the following necessary and sufficient conditions.

**Proposition** **1** (Necessary and sufficient conditions for narrow-sense group covariant signals [27])**.** *A set of quantum state signals $\left\{|\psi_{i}\rangle |i\in G\right\}$ is narrow-sense group covariant with respect to (G;∘) if and only if the following is the case.*

(22) $$\begin{array}{c} \forall i,j,k\in G,\;\;\langle \psi_{k\circ i}|\psi_{k\circ j}\rangle =\langle \psi_{i}|\psi_{j}\rangle . \end{array}$$

From this proposition, we can easily show that signals such as arbitrary binary pure-state signals and arbitrary *M*-ary PSK coherent-state signals are narrow-sense group covariant. In addition, for narrow-sense group covariant signals, analytic solutions for the eigenvalues and eigenvectors of the Gram matrix have been presented, indicating that narrow-sense group covariant signals are very useful for communication and cipher systems. In this study, we apply this knowledge to QAM signals that are not group-covariant.

## 3. Eigenvalues and Eigenvectors of M=4m-ary QAM Signals and Their Gram Matrix

In this section, we consider the eigenvalues and eigenvectors of the Gram matrix corresponding to M=4m-ary QAM signals. First, M=4m-ary QAM signals are defined and the corresponding Gram matrix is explained. Next, we state that the Gram matrix can be block-partitioned and clarify that it has the structure of the sum of tensor products. Finally, we show that the scale of the computation can be reduced.

### 3.1. 4m-ary QAM Signals

**Figure 1:**
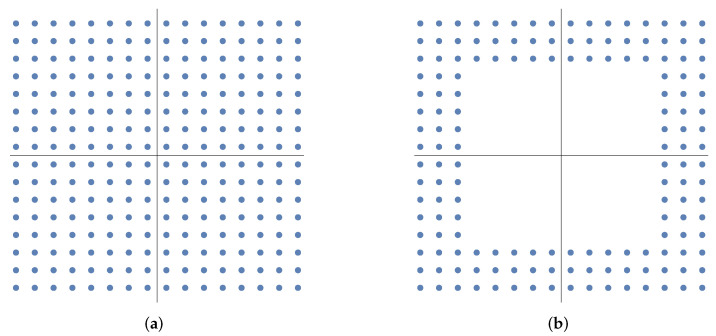
Examples of QAM signals presented in [29]. (**a**) 256QAM. (**b**) Modified 156QAM.

This subsection describes the 4m-ary QAM signals treated in this paper. QAM is a major modulation scheme used in digital communication, such as for coherent optical communication [40], and QAM signals are important for applications in quantum technologies such as quantum communication and quantum ciphers. Ordinary QAM signals are placed in a square lattice on the phase plane. As an example, Figure 1a shows the signal constellation of 256QAM on the phase plane. In quantum ciphers, modified QAM signals in which signals near the origin are removed have been proposed for higher security [29]. As an example, Figure 1b shows the signal constellation of the modified 156QAM on the phase plane. For 256QAM signals, the number of signals is M=4m=256 and $m=64=8^{2}$, while for modified 156QAM signals, it is M=4m=156 and m=39.

In this paper, we consider the signals defined below, which include both the ordinary QAM of Figure 1a and the modified QAM of Figure 1b, and we call them QAM signals.

**Definition** **2** (4m-ary QAM Signals)**.** *Let $\left\{\beta_{1},\beta_{2},\ldots ,\beta_{m}\right\}$ be any m-ary set of complex amplitudes for which its arguments lie in the range $0<\varphi <\frac{\pi }{2}$. That is, the complex amplitudes correspond to points in the first quadrant. Here, $\beta_{k}\neq 0\;\left(k=1,2,\ldots ,m\right)$ and $\beta_{k}\neq \beta_{k^{\prime }}\;\left(k\neq k^{\prime }\right)$ are assumed. For each $\beta_{k}$, let $\alpha_{k}^{\left(1\right)}=\beta_{k}$, $\alpha_{k}^{\left(2\right)}=i\beta_{k}$, $\alpha_{k}^{\left(3\right)}=-\beta_{k}$, and $\alpha_{k}^{\left(4\right)}=-i\beta_{k}$, where $i=\sqrt{-1}$. Then, we call the following set of coherent states “4m-ary QAM coherent-state signals” (4m-ary QAM signals for short):*

(23) $$S=\overset{m}{\underset{k=1}{⋃}}S_{k},$$

*where $S_{k}$ are sets of coherent states defined as follows.*

(24) $$S_{k}=\left\{|\alpha_{k}^{\left(i\right)}\rangle \;|\;i=1,2,3,4\right\}.$$

The rotation operator [4] that rotates the phase by an angle θ in the phase plane is represented as follows:

(25) $$\begin{array}{c} \hat{U}\left(\theta \right)=\exp\left[i\theta {\hat{a}}^{†}\hat{a}\right], \end{array}$$

where $\hat{a}$ and ${\hat{a}}^{†}$ are photon annihilation and creation operators, respectively. Rewriting $\hat{U}\left(\theta =\frac{\pi }{2}\right)$ as simply $\hat{U}$, $S_{k}$ becomes the following.

(26) $$\begin{array}{c} S_{k}=\left\{|\alpha_{k}^{\left(1\right)}\rangle ,\hat{U}|\alpha_{k}^{\left(1\right)}\rangle ,{\hat{U}}^{2}|\alpha_{k}^{\left(1\right)}\rangle ,{\hat{U}}^{3}|\alpha_{k}^{\left(1\right)}\rangle \right\}=\left\{|\beta_{k}\rangle ,\hat{U}|\beta_{k}\rangle ,{\hat{U}}^{2}|\beta_{k}\rangle ,{\hat{U}}^{3}|\beta_{k}\rangle \right\}. \end{array}$$

Here, we have the following.

(27) $$\begin{array}{c} {\hat{U}}^{4}={\hat{U}}^{0}=\hat{I}. \end{array}$$

The 4m-ary QAM signals defined above obviously include both ordinary QAM signals (e.g., Figure 1a) and modified QAM signals (e.g., Figure 1b). Although 4m-ary QAM signals are not symmetric signals, each subset $S_{k}$ is symmetric, group covariant, and geometrical uniform symmetric (GUS). Moreover, we should mention that 4m-ary QAM signals in Definition 2 satisfy the definition of the multiple constellations of GUS state [20], which is a particularization of the concept of compound geometrical uniform (CGU) states [41]. Hence, 4m-ary QAM signals are practical examples of the multiple constellations of GUS state and CGU states. The following results are also applicable when considering non-coherent states $|\psi_{k}\rangle$, such as squeezed states, instead of $|\beta_{k}\rangle$ in Equation (26).

### 3.2. Gram Matrix of 4m-ary QAM Signals

As shown in Equation (23), 4m-ary QAM signals are partitioned into *m* subsets $S_{k}\;\left(k=1,2,\ldots ,m\right)$. Let $\Gamma_{k,l}^{\left(4\right)}$ be the 4×4 matrix for which its entries are the inner product between two signals, where one of the two signals is chosen from the subset $S_{k}$, and the other is chosen from the subset $S_{l}$. Then, the Gram matrix of the 4m-ary QAM signals can be represented in block-partitioned form as follows.

(28) $$\begin{array}{c} \Gamma =\left[\begin{array}{cccc} \Gamma_{1,1}^{\left(4\right)} & \Gamma_{1,2}^{\left(4\right)} & \ldots & \Gamma_{1,m}^{\left(4\right)}\\ \Gamma_{2,1}^{\left(4\right)} & \Gamma_{2,2}^{\left(4\right)} & \ldots & \Gamma_{2,m}^{\left(4\right)}\\ \vdots & \vdots & \ddots & \vdots \\ \Gamma_{m,1}^{\left(4\right)} & \Gamma_{m,2}^{\left(4\right)} & \ldots & \Gamma_{m,m}^{\left(4\right)} \end{array}\right]. \end{array}$$

From Equation (26), the (i,j)-th element of $\Gamma_{k,l}^{\left(4\right)}$ is as follows.

(29) $$\begin{array}{ccc} {\left(\Gamma_{k,l}^{\left(4\right)}\right)}_{i,j} & = & \langle \alpha_{k}^{\left(1\right)}|{\left({\hat{U}}^{i-1}\right)}^{†}{\hat{U}}^{j-1}|\alpha_{l}^{\left(1\right)}\rangle =\langle \alpha_{k}^{\left(1\right)}|{\hat{U}}^{j-i}|\alpha_{l}^{\left(1\right)}\rangle =\langle \beta_{k}|{\hat{U}}^{j-i}|\beta_{l}\rangle . \end{array}$$

This implies that $\Gamma_{k,l}^{\left(4\right)}$ is cyclic.

Denoting the components of the first row of $\Gamma_{k,l}^{\left(4\right)}$ as $a_{k,l},b_{k,l},c_{k,l}$ and $d_{k,l}$, the submatrix $\Gamma_{k,l}^{\left(4\right)}$ is described as follows:

(30) $$\begin{array}{c} \Gamma_{k,l}^{\left(4\right)}=\left[\begin{array}{cccc} a_{k,l} & b_{k,l} & c_{k,l} & d_{k,l}\\ d_{k,l} & a_{k,l} & b_{k,l} & c_{k,l}\\ c_{k,l} & d_{k,l} & a_{k,l} & b_{k,l}\\ b_{k,l} & c_{k,l} & d_{k,l} & a_{k,l} \end{array}\right], \end{array}$$

where the following is the case.

$$\begin{array}{cc} \; & a_{k,l}=\langle \beta_{k}|\beta_{l}\rangle ,\\ \; & b_{k,l}=\langle \beta_{k}|\hat{U}|\beta_{l}\rangle =\langle \beta_{k}|i\beta_{l}\rangle ,\\ \; & c_{k,l}=\langle \beta_{k}|{\hat{U}}^{2}|\beta_{l}\rangle =\langle \beta_{k}|-\beta_{l}\rangle ,\\ \; & d_{k,l}=\langle \beta_{k}|{\hat{U}}^{3}|\beta_{l}\rangle =\langle \beta_{k}|-i\beta_{l}\rangle . \end{array}$$

### 3.3. Decomposition of Submatrices

Here, we consider the common properties of each $\Gamma_{k,l}^{\left(4\right)}$ by performing a spectral decomposition of each submatrix $\Gamma_{k,l}^{\left(4\right)}$ introduced in the previous section. Because $\Gamma_{k,l}^{\left(4\right)}$ is cyclic according to Equation (30), the analytic expressions of its eigenvalues $\lambda_{i}^{\left(k,l\right)}$ and eigenvectors $\lambda_{i}\;\left(i=1,2,3,4\right)$ are well known. The expressions are as follows.

$$\begin{array}{cc} \lambda_{1}^{\left(k,l\right)} & =a_{k,l}+b_{k,l}+c_{k,l}+d_{k,l},\\ \lambda_{2}^{\left(k,l\right)} & =a_{k,l}-b_{k,l}+c_{k,l}-d_{k,l},\\ \lambda_{3}^{\left(k,l\right)} & =a_{k,l}+ib_{k,l}-c_{k,l}-id_{k,l},\\ \lambda_{4}^{\left(k,l\right)} & =a_{k,l}-ib_{k,l}-c_{k,l}+id_{k,l}, \end{array}$$

$$\begin{array}{c} \lambda_{1}=\frac{1}{2}\left[\begin{array}{c} 1\\ 1\\ 1\\ 1 \end{array}\right],\;\lambda_{2}=\frac{1}{2}\left[\begin{array}{c} 1\\ -1\\ 1\\ -1 \end{array}\right],\;\lambda_{3}=\frac{1}{2}\left[\begin{array}{c} 1\\ i\\ -1\\ -i \end{array}\right],\;\lambda_{4}=\frac{1}{2}\left[\begin{array}{c} 1\\ -i\\ -1\\ i \end{array}\right]. \end{array}$$

As eigenvectors $\left\{\lambda_{1},\lambda_{2},\lambda_{3},\lambda_{4}\right\}$ are orthonormal, $\Gamma_{k,l}^{\left(4\right)}$ can be spectrally decomposed as follows:

(31) $$\begin{array}{c} \Gamma_{k,l}^{\left(4\right)}=\sum_{i=1}^{4}\lambda_{i}^{\left(k,l\right)}\lambda_{i}{\lambda_{i}}^{H}, \end{array}$$

where ${\lambda_{i}}^{H}$ denotes the conjugate transpose of $\lambda_{i}$.

### 3.4. Decomposition of Gram Matrix

In this subsection, we decompose the Gram matrix Γ into a sum of tensor products using the spectral decomposition of submatrices $\Gamma_{k,l}^{\left(4\right)}$. All $\Gamma_{k,l}^{\left(4\right)}$ have common eigenvectors independent of *k* and *l*. Substituting Equation (31) into Equation (28), we obtain the following:

(32) $$\begin{array}{cc} \Gamma & =\left[\begin{array}{cccc} \Gamma_{1,1}^{\left(4\right)} & \Gamma_{1,2}^{\left(4\right)} & \ldots & \Gamma_{1,m}^{\left(4\right)}\\ \Gamma_{2,1}^{\left(4\right)} & \Gamma_{2,2}^{\left(4\right)} & \ldots & \Gamma_{2,m}^{\left(4\right)}\\ \vdots & \vdots & \ddots & \vdots \\ \Gamma_{m,1}^{\left(4\right)} & \Gamma_{m,2}^{\left(4\right)} & \ldots & \Gamma_{m,m}^{\left(4\right)} \end{array}\right]\\ \; & =\sum_{i=1}^{4}\left[\begin{array}{cccc} \lambda_{i}^{\left(1,1\right)} & \lambda_{i}^{\left(1,2\right)} & \ldots & \lambda_{i}^{\left(1,m\right)}\\ \lambda_{i}^{\left(2,1\right)} & \lambda_{i}^{\left(2,2\right)} & \ldots & \lambda_{i}^{\left(2,m\right)}\\ \vdots & \vdots & \ddots & \vdots \\ \lambda_{i}^{\left(m,1\right)} & \lambda_{i}^{\left(m,2\right)} & \ldots & \lambda_{i}^{\left(m,m\right)} \end{array}\right]⊗\lambda_{i}{\lambda_{i}}^{H}\\ \; & =\sum_{i=1}^{4}A_{i}⊗\lambda_{i}{\lambda_{i}}^{H}, \end{array}$$

where the following is the case.

(33) $$\begin{array}{cc} A_{i} & =\left[\begin{array}{cccc} \lambda_{i}^{\left(1,1\right)} & \lambda_{i}^{\left(1,2\right)} & \ldots & \lambda_{i}^{\left(1,m\right)}\\ \lambda_{i}^{\left(2,1\right)} & \lambda_{i}^{\left(2,2\right)} & \ldots & \lambda_{i}^{\left(2,m\right)}\\ \vdots & \vdots & \ddots & \vdots \\ \lambda_{i}^{\left(m,1\right)} & \lambda_{i}^{\left(m,2\right)} & \ldots & \lambda_{i}^{\left(m,m\right)} \end{array}\right]. \end{array}$$

In the following, we show that each matrix $A_{i}$ consisting of the eigenvalues of $\Gamma_{k,l}^{\left(4\right)}$ is Hermitian. As Γ is the Gram matrix (and is therefore Hermitian), its submatrices satisfy the following.

(34) $$\begin{array}{c} {\left(\Gamma_{k,l}^{\left(4\right)}\right)}^{H}=\Gamma_{l,k}^{\left(4\right)}. \end{array}$$

From Equation (31), we have the following:

(35) $$\begin{array}{c} {\left(\Gamma_{k,l}^{\left(4\right)}\right)}^{H}=\sum_{i=1}^{4}{\left(\lambda_{i}^{\left(k,l\right)}\right)}^{*}\lambda_{i}{\lambda_{i}}^{H} \end{array}$$

and from Equation (34), it coincides with the following.

(36) $$\begin{array}{c} \Gamma_{l,k}^{\left(4\right)}=\sum_{i=1}^{4}\lambda_{i}^{\left(l,k\right)}\lambda_{i}{\lambda_{i}}^{H}. \end{array}$$

Thus, we have

(37) $$\begin{array}{c} {\left(\lambda_{i}^{\left(k,l\right)}\right)}^{*}=\lambda_{i}^{\left(l,k\right)}. \end{array}$$

Hence, all $A_{i}$ of Equation (33) are Hermitian.

(38) $$\begin{array}{c} {A_{i}}^{H}=A_{i},\;i\in \left\{1,2,3,4\right\}. \end{array}$$

Therefore, each $A_{i}$ is spectrally decomposable. Let $a_{j}^{\left(i\right)}$ and $a_{j}^{\left(i\right)}$ be the eigenvalues and corresponding orthonormalized eigenvectors of $A_{i}$. Then, the spectral decomposition form of $A_{i}$ is as follows.

(39) $$\begin{array}{c} A_{i}=\sum_{j=1}^{m}a_{j}^{\left(i\right)}a_{j}^{\left(i\right)}{a_{j}^{\left(i\right)}}^{H}. \end{array}$$

Substituting this into Equation (32), we obtain the following.

(40) $$\begin{array}{c} \Gamma =\sum_{i=1}^{4}\sum_{j=1}^{m}a_{j}^{\left(i\right)}a_{j}^{\left(i\right)}{a_{j}^{\left(i\right)}}^{H}⊗\lambda_{i}{\lambda_{i}}^{H}. \end{array}$$

### 3.5. Eigenvalues and Eigenvectors of Gram Matrix

In this subsection, we derive the eigenvalues and eigenvectors from the decomposition form (40) of the Gram matrix Γ. Because both $\left\{a_{j}^{\left(i\right)}\right\}$ and $\left\{\lambda_{i}\right\}$ are orthonormal, we have the following:

$$\begin{array}{c} \Gamma \left(a_{j}^{\left(i\right)}⊗\lambda_{i}\right)=a_{j}^{\left(i\right)}\left(a_{j}^{\left(i\right)}⊗\lambda_{i}\right)\;\left(j=1,\ldots ,m,\;i=1,2,3,4\right), \end{array}$$

and the eigenvalues and eigenvectors of the Gram matrix Γ of M=4m-ary QAM signals are listed in Table 1.

Therefore, to compute the eigenvalues and eigenvectors of the 4m×4m matrix Γ, it is sufficient to consider the eigenvalue problem of the smaller matrices $A_{i}\;\left(i=1,2,3,4\right)$.

### 3.6. Relation of the Results in the Relevant Literature

In this subsection, we consider the relation between the results in this paper and those in Ref. [20]. As examples of the multiple constellations of GUS state, the new signals were introduced [20]. They are called a double quantum binary phase shift keying (BPSK) and a double quantum pulse position modulation (PPM). As mentioned in Section 3.1, 4m-ary QAM signals also belong to the class of the multiple constellations of GUS state. The signals are not new, but they are rather traditional, and they are well known to be useful. Therefore, it is worth noticing that the results in Ref. [20] are also applicable to 4m-ary QAM signals. The most significant result is the optimality of SRM. That is, SRM can be an optimal measurement for 4m-ary QAM signals with certain *a priori* probabilities. Furthermore, various results had been obtained in Ref. [20] while they had shown the optimality of SRM. They provided the block-partitioned form of the Gram matrix and showed that each submatrix is diagonalizable by the Fourier matrix. These results correspond to the results in Section 3.2 and Section 3.3. Then, they considered a transformation of the matrix block-partitioned by diagonal submatrices into a block diagonal matrix. This result is closely related to the result in Section 3.4. Although they had not mentioned the eigenvalues and eigenvectors, one may connect their discussion for the square-root of the Gram matrix to the results in this section. We would like to emphasize here a reduction in computational costs, whereas they did not explicitly state a reduction.

## 4. Examples for the Case of m=2

Here, we consider the simplest case of m=2 as examples.

### 4.1. Submatrices $A_{i}$

From Equation (33), each $A_{i}$ consists of the eigenvalues $\lambda_{i}^{\left(k,l\right)}$ of $\Gamma_{\left(k,l\right)}^{\left(4\right)}$. Since $\lambda_{i}^{\left(k,l\right)}$ is a weighted sum of the inner products $\langle \beta_{k}|\beta_{l}\rangle ,\langle \beta_{k}|i\beta_{l}\rangle ,\langle \beta_{k}|-\beta_{l}\rangle$, and $\langle \beta_{k}|-i\beta_{l}\rangle$, it is convenient to describe the forms of the inner product for coherent states by using Equation (20):

$$\begin{array}{c} \langle \alpha |\pm \beta \rangle =e^{\pm \alpha^{*}\beta }e^{-\frac{{\left|\alpha \right|}^{2}}{2}-\frac{{\left|\beta \right|}^{2}}{2}},\;\langle \alpha |\pm i\beta \rangle =e^{\pm i\alpha^{*}\beta }e^{-\frac{{\left|\alpha \right|}^{2}}{2}-\frac{{\left|\beta \right|}^{2}}{2}}, \end{array}$$

where we set $\alpha =\beta_{k}$ and $\beta =\beta_{l}$. Using the above forms, we have the following:

(41) $$\begin{array}{ccc} \lambda_{1}^{\left(k,l\right)} & = & \langle \alpha |\beta \rangle +\langle \alpha |i\beta \rangle +\langle \alpha |-\beta \rangle +\langle \alpha |-i\beta \rangle \\ & = & \left(e^{\alpha^{*}\beta }+e^{i\alpha^{*}\beta }+e^{-\alpha^{*}\beta }+e^{-i\alpha^{*}\beta }\right)e^{-\frac{{\left|\alpha \right|}^{2}}{2}-\frac{{\left|\beta \right|}^{2}}{2}}\\ & = & 2\left\{\cosh\left(\alpha^{*}\beta \right)+\cos\left(\alpha^{*}\beta \right)\right\}e^{-\frac{{\left|\alpha \right|}^{2}}{2}-\frac{{\left|\beta \right|}^{2}}{2}}, \end{array}$$

(42) $$\begin{array}{ccc} \lambda_{2}^{\left(k,l\right)} & = & \langle \alpha |\beta \rangle -\langle \alpha |i\beta \rangle +\langle \alpha |-\beta \rangle -\langle \alpha |-i\beta \rangle \\ & = & 2\left\{\cosh\left(\alpha^{*}\beta \right)-\cos\left(\alpha^{*}\beta \right)\right\}e^{-\frac{{\left|\alpha \right|}^{2}}{2}-\frac{{\left|\beta \right|}^{2}}{2}}, \end{array}$$

(43) $$\begin{array}{ccc} \lambda_{3}^{\left(k,l\right)} & = & \langle \alpha |\beta \rangle +i\langle \alpha |i\beta \rangle -\langle \alpha |-\beta \rangle -i\langle \alpha |-i\beta \rangle \\ & = & 2\left\{\sinh\left(\alpha^{*}\beta \right)-\sin\left(\alpha^{*}\beta \right)\right\}e^{-\frac{{\left|\alpha \right|}^{2}}{2}-\frac{{\left|\beta \right|}^{2}}{2}}, \end{array}$$

(44) $$\begin{array}{ccc} \lambda_{4}^{\left(k,l\right)} & = & \langle \alpha |\beta \rangle -i\langle \alpha |i\beta \rangle -\langle \alpha |-\beta \rangle +i\langle \alpha |-i\beta \rangle \\ & = & 2\left\{\sinh\left(\alpha^{*}\beta \right)+\sin\left(\alpha^{*}\beta \right)\right\}e^{-\frac{{\left|\alpha \right|}^{2}}{2}-\frac{{\left|\beta \right|}^{2}}{2}}, \end{array}$$

where we write the following.

$$\begin{array}{c} \frac{e^{x}+e^{-x}}{2}=\cosh\left(x\right),\;\frac{e^{ix}+e^{-ix}}{2}=\cos\left(x\right),\\ \frac{e^{x}-e^{-x}}{2}=\sinh\left(x\right),\;\frac{e^{ix}-e^{-ix}}{2i}=\sin\left(x\right). \end{array}$$

From Equations (41)–(44), we obtain for the case of m=2:

(45) $$\begin{array}{ccc} A_{1} & = & \left[\begin{array}{cc} \cosh(|\beta_{1}{|}^{2})+\cos(|\beta_{1}{|}^{2}) & \cosh\left(\beta_{1}^{*}\beta_{2}\right)+\cos\left(\beta_{1}^{*}\beta_{2}\right)\\ \cosh\left(\beta_{2}^{*}\beta_{1}\right)+\cos\left(\beta_{2}^{*}\beta_{1}\right) & \cosh(|\beta_{2}{|}^{2})+\cos(|\beta_{2}{|}^{2}) \end{array}\right]\circ \left(2X\right), \end{array}$$

(46) $$\begin{array}{ccc} A_{2} & = & \left[\begin{array}{cc} \cosh(|\beta_{1}{|}^{2})-\cos(|\beta_{1}{|}^{2}) & \cosh\left(\beta_{1}^{*}\beta_{2}\right)-\cos\left(\beta_{1}^{*}\beta_{2}\right)\\ \cosh\left(\beta_{2}^{*}\beta_{1}\right)-\cos\left(\beta_{2}^{*}\beta_{1}\right) & \cosh(|\beta_{2}{|}^{2})-\cos(|\beta_{2}{|}^{2}) \end{array}\right]\circ \left(2X\right), \end{array}$$

(47) $$\begin{array}{ccc} A_{3} & = & \left[\begin{array}{cc} \sinh(|\beta_{1}{|}^{2})-\sin(|\beta_{1}{|}^{2}) & \sinh\left(\beta_{1}^{*}\beta_{2}\right)-\sin\left(\beta_{1}^{*}\beta_{2}\right)\\ \sinh\left(\beta_{2}^{*}\beta_{1}\right)-\sin\left(\beta_{2}^{*}\beta_{1}\right) & \sinh(|\beta_{2}{|}^{2})-\sin(|\beta_{2}{|}^{2}) \end{array}\right]\circ \left(2X\right), \end{array}$$

(48) $$\begin{array}{ccc} A_{4} & = & \left[\begin{array}{cc} \sinh(|\beta_{1}{|}^{2})+\sin(|\beta_{1}{|}^{2}) & \sinh\left(\beta_{1}^{*}\beta_{2}\right)+\sin\left(\beta_{1}^{*}\beta_{2}\right)\\ \sinh\left(\beta_{2}^{*}\beta_{1}\right)+\sin\left(\beta_{2}^{*}\beta_{1}\right) & \sinh(|\beta_{2}{|}^{2})+\sin(|\beta_{2}{|}^{2}) \end{array}\right]\circ \left(2X\right), \end{array}$$

where ∘ denotes the Hadamard product, and the following is the case.

(49) $$\begin{array}{c} X=\left[\begin{array}{cc} e^{-|\beta_{1}{|}^{2}} & e^{-\frac{|\beta_{1}{|}^{2}}{2}-\frac{|\beta_{2}{|}^{2}}{2}}\\ e^{-\frac{|\beta_{1}{|}^{2}}{2}-\frac{|\beta_{2}{|}^{2}}{2}} & e^{-|\beta_{2}{|}^{2}} \end{array}\right]. \end{array}$$

The remaining task is to calculate the eigenvalues and eigenvectors of the 2×2 matrices $A_{1}$∼$A_{4}$. Although it is possible to calculate eigenvalues and eigenvectors of a 2×2 matrix, the general form may be slightly complicated. In the following, we consider simple two cases.

### 4.2. Case of $|\beta_{1}\left|=\right|\beta_{2}|=\gamma$

This case corresponds to phase-mismatching PSK signals. The signals are similar to the double quantum BPSK with a misalignment or a systematic bias error in the angle defining one of the two constellations [20]. Note that the number of signals is different. In this case, from the following:

$$\begin{array}{c} X=\left[\begin{array}{cc} e^{-\gamma^{2}} & e^{-\gamma^{2}}\\ e^{-\gamma^{2}} & e^{-\gamma^{2}} \end{array}\right], \end{array}$$

“∘(2X)” in Equations (45)–(48) becomes simply a scalar product “$\times (2e^{-\gamma^{2}})$”. Therefore, each $A_{i}$ has the following form:

$$\begin{array}{c} \left[\begin{array}{cc} a & b\\ b^{*} & a \end{array}\right], \end{array}$$

where *a* is a real number and *b* is a complex number. The eigenvalues and the corresponding orthonormal eigenvectors of the above form are the following:

(50) $$\begin{array}{c} a\pm |b|,\;\frac{1}{\sqrt{2}}\left[\begin{array}{c} 1\\ \pm e^{-i\mu } \end{array}\right], \end{array}$$

where μ=arg(b). Therefore, the eigenvalues of $A_{i}$ are as follows:

(51) $$\begin{array}{ccc} a_{1}^{\left(1\right)} & = & 2e^{-\gamma^{2}}\left\{\cosh\left(\gamma^{2}\right)+\cos\left(\gamma^{2}\right)+\left|\cosh(\delta )+\cos(\delta )\right|\right\}, \end{array}$$

(52) $$\begin{array}{ccc} a_{2}^{\left(1\right)} & = & 2e^{-\gamma^{2}}\left\{\cosh\left(\gamma^{2}\right)+\cos\left(\gamma^{2}\right)-\left|\cosh(\delta )+\cos(\delta )\right|\right\}, \end{array}$$

(53) $$\begin{array}{ccc} a_{1}^{\left(2\right)} & = & 2e^{-\gamma^{2}}\left\{\cosh\left(\gamma^{2}\right)-\cos\left(\gamma^{2}\right)+\left|\cosh(\delta )-\cos(\delta )\right|\right\}, \end{array}$$

(54) $$\begin{array}{ccc} a_{2}^{\left(2\right)} & = & 2e^{-\gamma^{2}}\left\{\cosh\left(\gamma^{2}\right)-\cos\left(\gamma^{2}\right)-\left|\cosh(\delta )-\cos(\delta )\right|\right\}, \end{array}$$

(55) $$\begin{array}{ccc} a_{1}^{\left(3\right)} & = & 2e^{-\gamma^{2}}\left\{\sinh\left(\gamma^{2}\right)-\sin\left(\gamma^{2}\right)+\left|\sinh(\delta )-\sin(\delta )\right|\right\}, \end{array}$$

(56) $$\begin{array}{ccc} a_{2}^{\left(3\right)} & = & 2e^{-\gamma^{2}}\left\{\sinh\left(\gamma^{2}\right)-\sin\left(\gamma^{2}\right)-\left|\sinh(\delta )-\sin(\delta )\right|\right\}, \end{array}$$

(57) $$\begin{array}{ccc} a_{1}^{\left(4\right)} & = & 2e^{-\gamma^{2}}\left\{\sinh\left(\gamma^{2}\right)+\sin\left(\gamma^{2}\right)+\left|\sinh(\delta )+\sin(\delta )\right|\right\}, \end{array}$$

(58) $$\begin{array}{ccc} a_{2}^{\left(4\right)} & = & 2e^{-\gamma^{2}}\left\{\sinh\left(\gamma^{2}\right)+\sin\left(\gamma^{2}\right)-\left|\sinh(\delta )+\sin(\delta )\right|\right\}, \end{array}$$

where we set $\beta_{1}=\gamma e^{\nu_{1}},\beta_{2}=\gamma e^{\nu_{2}},\beta_{1}^{*}\beta_{2}=\gamma^{2}e^{i(\nu_{2}-\nu_{1})}=\delta$.

The eigenvectors of $A_{i}$ are as follows:

(59) $$\begin{array}{ccc} & a_{1}^{\left(i\right)}=\frac{1}{\sqrt{2}}\left[\begin{array}{c} 1\\ e^{-i\mu_{i}} \end{array}\right],\; & a_{2}^{\left(i\right)}=\frac{1}{\sqrt{2}}\left[\begin{array}{c} 1\\ -e^{-i\mu_{i}} \end{array}\right],\;\left(i=1,2,3,4\right) \end{array}$$

where the following is the case.

$$\begin{array}{ccc} & \mu_{1}=\arg\left(\cosh(\delta )+\cos(\delta )\right), & \mu_{2}=\arg\left(\cosh(\delta )-\cos(\delta )\right),\\ & \mu_{3}=\arg\left(\sinh(\delta )-\sin(\delta )\right), & \mu_{4}=\arg\left(\sinh(\delta )+\sin(\delta )\right). \end{array}$$

### 4.3. Case of $\arg\left(\beta_{1}\right)=\arg\left(\beta_{2}\right)=\nu$

The signals in this case are similar to the four-pulse amplitude modulation (PAM) [20]. Note that the number of signals is eight in this case, but four for the 4-PAM. In this case, the form of $A_{i}$ is as follows:

(60) $$\begin{array}{c} \left[\begin{array}{cc} a & b\\ b & c \end{array}\right], \end{array}$$

where a,b, and *c* are real numbers. The eigenvalues of the matrix with this form are as follows:

(61) $$\begin{array}{c} \frac{1}{2}\left(a+c\pm \sqrt{{\left(a-c\right)}^{2}+4b^{2}}\right), \end{array}$$

and the corresponding orthogonal eigenvectors are the following.

(62) $$\begin{array}{c} \left[\begin{array}{c} a-c\pm \sqrt{{\left(a-c\right)}^{2}+4b^{2}}\\ 2b \end{array}\right]. \end{array}$$

We obtain the orthonormal eigenvectors by normalizing them. Using the above equations, we can obtain the explicit forms of $a_{1}^{\left(1\right)}∼a_{2}^{\left(4\right)}$ and $a_{1}^{\left(1\right)}∼a_{2}^{\left(4\right)}$ as the same manner in Section 4.2.

## 5. Numerical Experiments

Here, we provide numerical experiments as examples of application for the results in Section 3. We consider 16QAM signals (the case of m=4) in this section. Set $\beta_{1}=\left(1+i\right)\alpha$, $\beta_{2}=\left(3+i\right)\alpha ,\beta_{3}=\left(3+3i\right)\alpha ,\beta_{4}=\left(1+3i\right)\alpha$. The average number of photons of 16QAM coherent-state signals is as follows:

$$\frac{1}{4}\left({\left|(1+i)\alpha \right|}^{2}+{\left|(3+i)\alpha \right|}^{2}+{\left|(3+3i)\alpha \right|}^{2}+{\left|(1+3i)\alpha \right|}^{2}\right)=10{\left|\alpha \right|}^{2},$$

and it is proportional to ${\left|\alpha \right|}^{2}$. Hence, in the following, we show numerical results of some quantities with respect to ${\left|\alpha \right|}^{2}$.

### 5.1. Von Neumann Entropy

First, we consider the von Neumann entropy, which is calculated by using eigenvalues of the Gram matrix. Since the Holevo capacity is the maximization of the von Neumann entropy with respect to *a priori* probabilities, the von Neumann entropy is a lower bound on the capacity. Let $\hat{\rho }$ be the density operator of 16QAM signals. Then, the von Neumann entropy (9) is calculated by the eigenvalues of $\hat{\rho }$ as follows.

$$\chi =-\sum_{j=1}^{16}\lambda_{j}\log_{2}\lambda_{j}.$$

Each $\lambda_{j}$ is equal to an eigenvalue of $\frac{1}{16}\Gamma$ from Equation (13). According to the results in Section 3, the following is the case:

$$\begin{array}{ccc} \chi & = & -\sum_{i=1}^{4}\sum_{j=1}^{4}\left(\frac{1}{16}a_{j}^{\left(i\right)}\right)\log_{2}\left(\frac{1}{16}a_{j}^{\left(i\right)}\right)=-\frac{1}{16}\sum_{i=1}^{4}\sum_{j=1}^{4}a_{j}^{\left(i\right)}\left(\log_{2}a_{j}^{\left(i\right)}-\log_{2}16\right)\\ & = & 4-\frac{1}{16}\sum_{i=1}^{4}\sum_{j=1}^{4}a_{j}^{\left(i\right)}\log_{2}a_{j}^{\left(i\right)}, \end{array}$$

where $a_{j}^{\left(i\right)}$ are eigenvalues of the matrices $A_{i}$ described in Section 3, and we numerically calculate $a_{j}^{\left(i\right)}$. Note that we only need numerical calculation of eigenvalues for smaller matrices $A_{i}$ than the original Gram matrix Γ.

**Figure 2:**
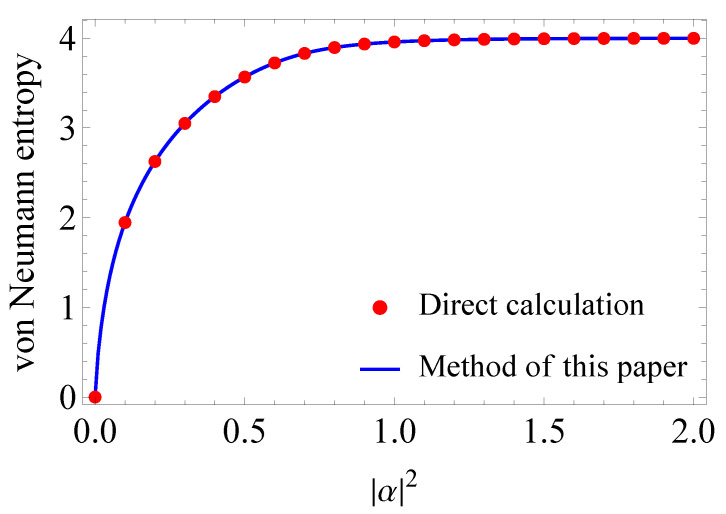
von Neumann entropy of 16QAM signals with respect to ${\left|\alpha \right|}^{2}$. The blue line is drawn by using the results in Section 3, while the red dots are plotted by using direct calculation of eigenvalues for the Gram matrix.

Figure 2 shows the von Neumann entropy of 16QAM signals with respect to ${\left|\alpha \right|}^{2}$. The blue line is drawn by using the results in Section 3, while the red dots are plotted by using direct calculation of eigenvalues for the Gram matrix. From Figure 2, we can confirm that both results are identical.

### 5.2. Error Probability

Now, we consider the error probability by using the SRM. To compute the error probability, both eigenvalues and eigenvectors of the Gram matrix are needed. As explained in Section 2, the error probability is as follows.

$$P_{e}=1-\frac{1}{16}\sum_{i=1}^{16}P\left(i|i\right)=1-\frac{1}{16}\sum_{i=1}^{16}{\left|{\left(\Gamma^{\frac{1}{2}}\right)}_{i,i}\right|}^{2}.$$

From Equation (40), we have the following.

$$\Gamma^{\frac{1}{2}}=\sum_{i=1}^{4}\sum_{j=1}^{4}\sqrt{a_{j}^{\left(i\right)}}a_{j}^{\left(i\right)}{a_{j}^{\left(i\right)}}^{H}⊗\lambda_{i}{\lambda_{i}}^{H}.$$

Then, numerically calculating the eigenvalues $a_{j}^{\left(i\right)}$ and the eigenvectors $a_{j}^{\left(i\right)}$ for matrices $A_{i}$, and substituting them into the above equation, we obtain the error probability.

**Figure 3:**
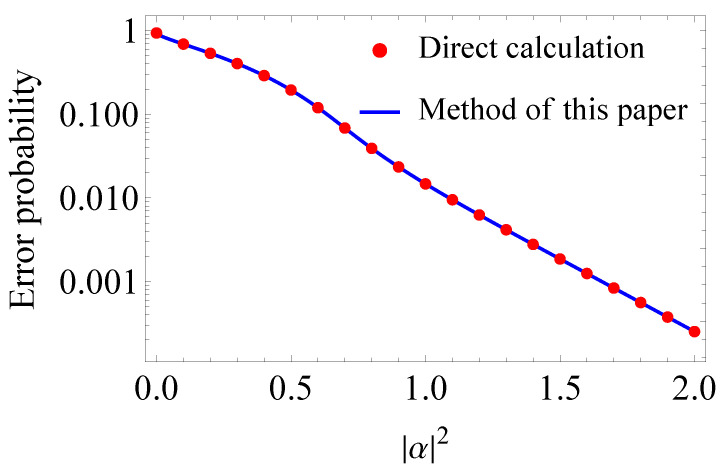
Error probability of 16QAM signals with respect to ${\left|\alpha \right|}^{2}$. The blue line is drawn by using the results in Section 3, while the red dots are plotted by using direct calculation of the matrix square-root for the Gram matrix.

Figure 3 shows the error probability of 16QAM signals with respect to ${\left|\alpha \right|}^{2}$. The blue line and the red dots have the same meaning as in Figure 2. From Figure 3, we can confirm that both results are identical.

## 6. Conclusions

In this paper, we have described the simplification of the Gram matrix eigenvalue problem for QAM coherent-state signals and shown that the scale of the computation can be reduced. As explained in Section 2, by solving the eigenvalue problem of the Gram matrix, it is possible to calculate quantities such as the error probability, mutual information, Holevo capacity, and the upper and lower bounds of the reliability function, which are important for evaluating the performance of quantum communication, quantum radar, and the security of quantum cryptography. The QAM signals treated in this study are very versatile, being applicable not only to ordinary QAM signals but also to any signals generated by rotation in the first quadrant of the phase plane. The quantum state used is typically but not necessarily the coherent state. In fact, the QAM signals defined in this paper belong to the class of the multiple constellations of GUS [20] and CGU states [41]. Therefore, the results in the literature are also applicable to QAM signals. Moreover, some results in Ref. [20] are closely related to the results in this paper, as explained in Section 3.6.

The most significant challenge for the future is the further simplification of the eigenvalue problem of the Gram matrix. For this purpose, the regularity of the signal constellation in the first quadrant of the phase plane should be taken into account. Therefore, carefully determining the order of signals in the first quadrant is important, even if they are the same signals. Another challenge is to apply the methods of this study to actual problems, whereas we have shown simple examples for 16QAM. For this purpose, the combined use of numerical algorithms (e.g., [42]) for the matrix calculations should be considered.

## Figures and Tables

**Table 1:** Eigenvalues and eigenvectors of Γ(j=1,…,m).

Eigenvalues	Eigenvectors
$a_{j}^{\left(1\right)}$	$a_{j}^{\left(1\right)}⊗\lambda_{1}$
$a_{j}^{\left(2\right)}$	$a_{j}^{\left(2\right)}⊗\lambda_{2}$
$a_{j}^{\left(3\right)}$	$a_{j}^{\left(3\right)}⊗\lambda_{3}$
$a_{j}^{\left(4\right)}$	$a_{j}^{\left(4\right)}⊗\lambda_{4}$

## Data Availability

Not applicable.

## Abbreviations

The following abbreviations are used in this manuscript:

SRM	Square-root measurement;
PSK	Phase shift keying;
ASK	Amplitude shift keying;
QAM	Quadrature amplitude modulation;
AMPM	Amplitude-modulated phase-modulated;
POVM	Positive operator-valued measure.
